# Synthesis and Characterization of Side-Chain Liquid-Crystalline Block Copolymers Containing Cyano-Terminated Phenyl Benzoate Moieties

**DOI:** 10.3390/molecules28237849

**Published:** 2023-11-29

**Authors:** Kaito Takahashi, Daisuke Taguchi, Takashi Kajitani, Takanori Fukushima, Shoichi Kubo, Atsushi Shishido

**Affiliations:** 1 Laboratory for Chemistry and Life Science, Institute of Innovative Research, Tokyo Institute of Technology, R1–12, 4259 Nagatsuta, Midori-ku, Yokohama 226-8501, Japan; 2 Department of Chemical Science and Engineering, School of Materials and Chemical Technology, Tokyo Institute of Technology, 2–12–1 Ookayama, Meguro-ku, Tokyo 152-8552, Japan; 3 TC College Promotion Office, Open Facility Center, Tokyo Institute of Technology, 4259 Nagatsuta, Midori-ku, Yokohama 226-8501, Japan; 4 Living Systems Materialogy (LiSM) Research Group, International Research Frontiers Initiative (IRFI), Tokyo Institute of Technology, 4259 Nagatsuta, Midori-ku, Yokohama 226-8501, Japan

**Keywords:** block copolymer, liquid crystal, microphase-separated structure, alignment

## Abstract

Block copolymers, known for their capacity to undergo microphase separation, spontaneously yield various periodic nanostructures. These precisely controlled nanostructures have attracted considerable interest due to their potential applications in microfabrication templates, conducting films, filter membranes, and other areas. However, it is crucial to acknowledge that microphase-separated structures typically exhibit random alignment, making alignment control a pivotal factor in functional material development. To address this challenge, researchers have explored the use of block copolymers containing liquid-crystalline (LC) polymers, which offer a promising technique for alignment control. The molecular structure and LC behavior of these polymers significantly impact the morphology and alignment of microphase-separated structures. In this study, we synthesized LC diblock copolymers with cyano-terminated phenyl benzoate moieties and evaluated the microphase-separated structures and molecular alignment behaviors. The LC diblock copolymers with a narrow molecular weight distribution were synthesized by atom transfer radical polymerization. Small angle X-ray scattering measurements revealed that the block copolymers exhibit smectic LC phases and form cylinder structures with a lattice period of about 18 nm by microphase separation. The examination of block copolymer films using polarized optical microscopy and polarized UV-visible absorption spectroscopy corroborated that the LC moieties were uniaxially aligned along the alignment treatment direction.

## 1. Introduction

Block copolymers, comprising covalently bonded multiple polymer chains, exhibit the spontaneous formation of periodic nanostructures through microphase separation. The specific morphologies, such as spheres, cylinders, lamellas, and gyroids, within these phase-separated structures depend on the volume ratio of the polymer blocks [1,2]. The allure of microphase-separated structures lies in their significant implications for fundamental science and industrial applications. Notably, they have been proposed for applications including microfabrication templates [3,4,5,6], conducting films [7,8], and filter membranes [9,10], capitalizing on the regularity and anisotropy of the structures. However, it is crucial to note that microphase-separated structures typically exhibit random alignment, underscoring the paramount importance of alignment control for the development of functional materials [11]. Directed self-assembly, involving substrates with guide patterns [12], has emerged as a fascinating technique to achieve fine alignment control. Notable methods include graphoepitaxy using physical concavo-convex guide patterns [13,14] and chemoepitaxy employing chemically surface-modified patterns [15,16,17]. Additionally, various alignment control methods have been reported, utilizing external stimuli, such as electric and magnetic fields, mechanical forces, and photo-irradiation [18,19,20,21,22,23].

Another alternative avenue for regulating microphase-separated structures involves the incorporation of liquid crystals (LCs) with inherent self-assembly capabilities [24]. LC molecules, possessing both fluidity and optical anisotropy, offer spontaneous alignment and responsiveness to external stimuli, rendering them valuable in the regulation of microphase-separated structures through molecular alignment control. For instance, block copolymers composed of a side-chain LC polymer with azobenzene moieties and poly(ethylene oxide) (PEO) have been explored [25,26,27,28]. The annealing process alone facilitated the formation of highly ordered cylinder-shaped PEO domains attributable to the smectic layers of the side-chain azobenzene mesogens. These vertically aligned PEO cylinders have been applied as ion channels or reaction fields, leading to the selective etching of substrates [29], the fabrication of metal nanodot arrays [30], conductive polymer nanowires [31], and more. The alignment control of the cylinder domains has also been achieved through surface treatment with alignment layers [32] or photoreaction with linearly polarized light [33,34,35]. Furthermore, the alignment direction of microphase-separated structures was reversibly altered using block copolymers comprising an LC polymer and polystyrene [36,37]. The impact of LC block copolymers on microphase separation has been studied in the context of various mesogens, including biphenyl and phenyl benzoate moieties [38,39,40,41,42,43].

The molecular structure of LC polymers within block copolymers exerts a significant influence on both the LC properties and the morphology of the microphase-separated structures. Hence, it becomes imperative to conduct in-depth investigations into the fundamental properties of LC block copolymers featuring diverse LC structures. We previously studied the microphase separation of nematic LC block copolymers consisting of methoxy-terminated phenyl benzoate moieties. In this study, we synthesized LC diblock copolymers consisting of smectic LC polymers with cyano-terminated phenyl benzoate moieties. They are expected to possess the flexibility of the phenyl benzoate moieties with the regularity of intermolecular interactions associated with the polarity of the cyano terminus. Their LC behavior, microphase-separated structures, and molecular alignment behavior were investigated.

## 2. Results and Discussion

### 2.1. Synthesis of LC Homopolymer and LC Diblock Copolymers with Cyano-Terminated Phenyl Benzoate Moieties

**Figure 1:**
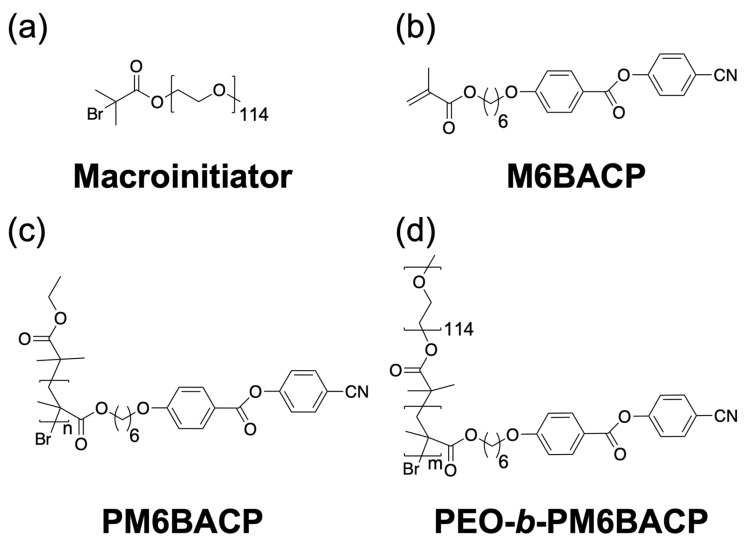
Chemical structures of the materials used in this study: (**a**) macroinitiator, (**b**) monomer M6BACP, (**c**) homopolymer PM6BACP, and (**d**) block copolymer PEO-*b*-PM6BACP.

**Figure 2:**
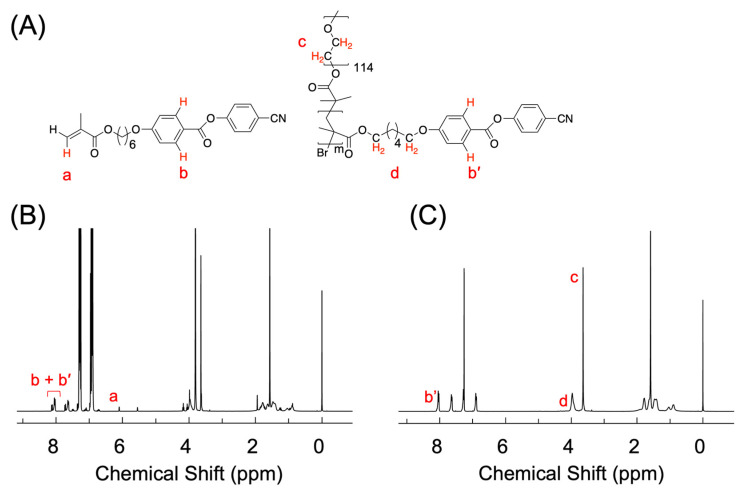
(**A**) Chemical structures representing protons used for ^1^H NMR analysis. (**B**,**C**) Typical ^1^H NMR spectra of PEO-*b*-PM6BACP_106_ in CDCl_3_ (**B**) before and (**C**) after purification. The peaks at 7.3, 6.9, and 3.7 ppm in (**A**) are derived from the residue solvent anisole. The labels a, b, b’, c, and d represent the positions of the protons (**A**) and corresponding peaks (**B**,**C**) used for the calculation of the monomer conversion and the degree of polymerization.

A homopolymer, namely poly(methacrylate) featuring side chains terminated with cyano-functionalized phenyl benzoate mesogens (referred to as PM6BACP) and a series of block copolymers denoted as PEO-*b*-PM6BACP (as illustrated in Figure 1) were successfully synthesized through the application of atom transfer radical polymerization (ATRP). The key characteristics of the synthesized polymers are concisely summarized in Table 1. The monomer conversion of unrefined samples following the polymerization process was ascertained through ^1^H NMR measurements (Figure 2B) based on the intensity of the peaks located at 8.0 ppm (*I*_8.0_) showing the two protons in the phenyl group of both the monomer and the polymer (b and b′) and at 6.1 ppm (*I*_6.1_) showing the vinyl proton in the methacrylate group of the monomer (a) (Equation (1)).

(1) $$Conv.\;\left(\%\right)=\left(I_{8.0}-2\times I_{6.1}\right)/I_{8.0}\times 100$$

The theoretical degree of polymerization (DP(calc)) was given by the conversion and feed ratio of the monomer to initiator (Equation (2)).

(2) $$DP\left(calc\right)={\left[M\right]}_{0}/{\left[I\right]}_{0}\times Conv.$$

The theoretical molecular weight (*M*_n_(calc)) was calculated based on DP(calc) using Equation (3) for a homopolymer and Equation (4) for block copolymers, where 195, 5230, and 408 are the molecular weight of the initiator, PEO macroinitiator, and monomer, respectively.

(3) $$M_{n}\left(calc\right)=195+{\left[M\right]}_{0}/{\left[I\right]}_{0}\times Conv.\times 408$$

(4) $$M_{n}\left(calc\right)=5230+{\left[M\right]}_{0}/{\left[I\right]}_{0}\times Conv.\times 408$$

The DP for the purified block copolymers was approximately evaluated by the ratio of the integration of the oxyethylene protons pertaining to the PEO block at 3.6 ppm (*I*_3.6_) (c in Figure 2C) to that of the oxymethylene protons in the PM6BACP block at 4.0 ppm (*I*_4.0_) (d in Figure 2C) measured by ^1^H NMR (Equation (5)). The DP of the PEO block with an *M*_n_ of 5230 was 114. The calculated number-average molecular weight based on ^1^H NMR (*M*_n_(NMR)) was then given by Equation (6).

(5) $$DP=114\times I_{4.0}/I_{3.6}$$

(6) $$M_{n}\left(NMR\right)=5230+DP\times 408$$

The DP was 46 and 106 for two types of PEO-*b*-PM6BACP. The *M*_n_(NMR) of PEO-*b*-PM6BACP_46_ and PEO-*b*-PM6BACP_106_ were 24,000 and 48,500, respectively. The weight fraction of the LC block calculated by Equation (7) was 78 for PEO-*b*-PM6BACP_46_ and 89 for PEO-*b*-PM6BACP_106_.

(7) $$Weight\;fraction\;of\;LC\;block\left(\%\right)=\mathrm{DP}\times 408/M_{n}\left(NMR\right)\times 100$$

**Figure 3:**
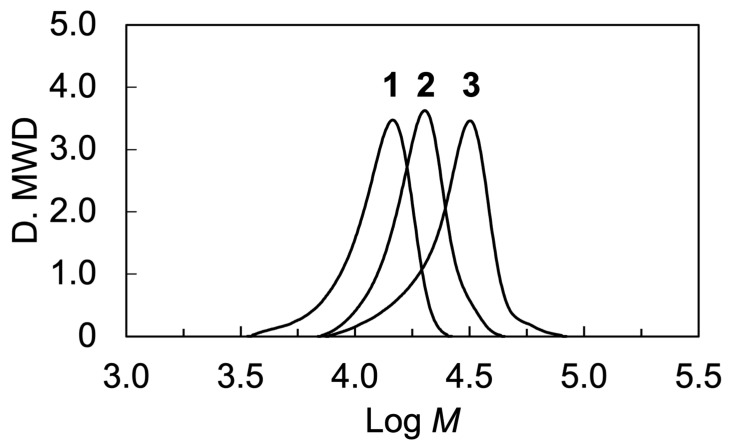
SEC curves of PM6BACP (**1**), PEO-*b*-PM6BACP_46_ (**2**), and PEO-*b*-PM6BACP_106_ (**3**).

Figure 3 shows size-exclusion chromatography (SEC) curves of the synthesized polymers. The number-average molecular weights (*M*_n_(SEC)) of PM6BACP, PEO-*b*-PM6BACP_46_, and PEO-*b*-PM6BACP_106_ were 11,800, 19,200, and 25,700. The difference in *M*_n_(SEC) of block copolymers is smaller than the *M*_n_(NMR). In a prior study involving the synthesis of block copolymers comprising PEO and side-chain LC polymers, similar disparities between the *M*_n_ values determined by SEC and those approximately evaluated through NMR measurements were documented [25]. Generally, SEC is not absolute but rather a relative analysis for molecular weights based on the calibration using standard samples. We consider that the small difference in *M*_n_(SEC) for the block copolymers is caused by the different chemical structures from polystyrene standards. The polydispersity of PM6BACP, PEO-*b*-PM6BACP_46_, and PEO-*b*-PM6BACP_106_ were 1.11, 1.09, and 1.13, respectively. Both the homopolymer and the block copolymers, all exhibiting a narrow molecular weight distribution of less than 1.15, were effectively synthesized via ATRP.

### 2.2. LC Properties

**Figure 4:**
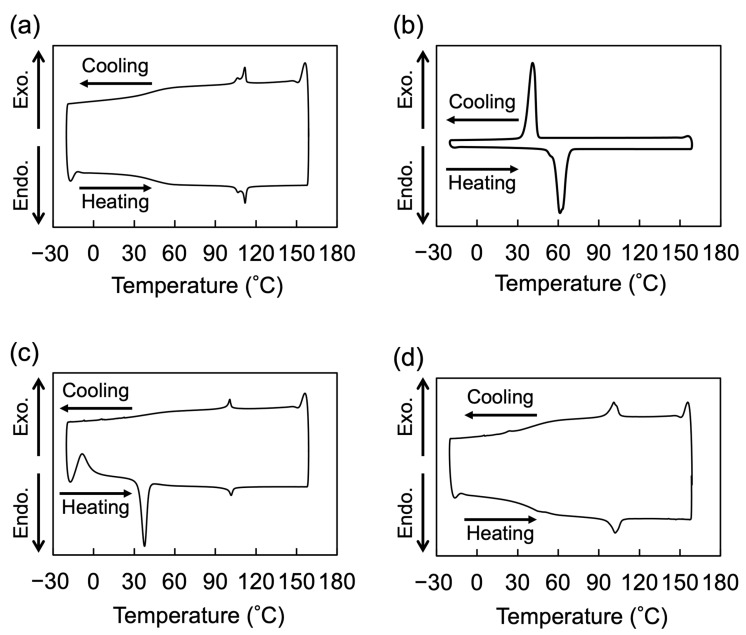
DSC thermograms of PM6BACP (**a**), PEO macroinitiator (**b**), PEO-*b*-PM6BACP_46_ (**c**), and PEO-*b*-PM6BACP_106_ (**d**) during the third heating and cooling processes at a scan rate of 10 °C/min.

**Figure 5:**
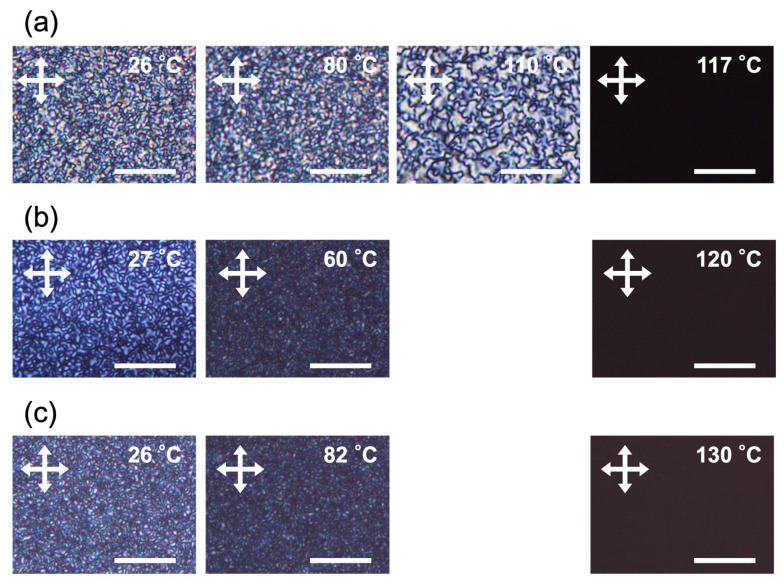
Polarized optical microscope images during the heating process of PM6BACP (**a**), PEO-*b*-PM6BACP_46_ (**b**), and PEO-*b*-PM6BACP_106_ (**c**). The samples were annealed at 120 °C for 10 min and gradually cooled to room temperature prior to the observation. Scale bars, 20 µm. White crossed arrows show the direction of the polarizers.

The LC properties of the polymers were examined using two methods: differential scanning calorimetry (DSC) and polarized optical microscopy (POM). In the case of the homopolymer PM6BACP, the DSC measurement revealed a baseline shift indicative of its glass transition temperature (*T*_g_) at 28 °C and endothermic peaks at 107 and 112 °C during the heating process (Figure 4a). As stated in a previous report [44], these observed peaks can be attributed to the transitions from the smectic to nematic phase and from the nematic to isotropic phase. Additionally, the formation of LC phases was further substantiated through POM under crossed polarizers. The optical textures were observed below 112 °C, while completely dark images were observed above 112 °C (Figure 5a). Under the observation condition in this study, almost the same textures except for the domain size were observed between 100 and 80 °C. The block copolymer PEO-*b*-PM6BACP_46_ showed an exothermic peak at −9 °C and endothermic peaks at 38 and 102 °C during the heating process (Figure 4c). Based on the thermograms of both the homopolymer and the PEO macroinitiator (as depicted in Figure 4b), it is evident that the presence of two endothermic peaks is linked to the melting of PEO and the transition from the LC phase to the isotropic phase of the PM6BACP block. Notably, during the cooling process, only a discernible exothermic peak, associated with the transition from the isotropic phase to the LC phase was observed at 101 °C. It is indicated that PEO was supercooled until −20 °C, and the crystallization occurred during the heating process at −9 °C. On the other hand, PEO-*b*-PMA6BACP_106_ showed an endothermic peak at 102 °C and an exothermic peak at 101 °C derived from LC to isotropic phase transition during heating and cooling processes, respectively (Figure 4d). The small endothermic peak at 49 °C and exothermic peak at 24 °C during the heating and cooling processes, respectively, are attributed to the melt and crystallization of PEO. The significantly small peaks suggest that the LC property was dominant because of the large fraction of the LC block in the block copolymer, and the crystallization of PEO was suppressed. The optical textures observed by POM under crossed polarizers confirmed LC phases of both the block copolymers below the phase transition temperatures (Figure 5b,c). POM observations conducted at room temperature for both block copolymers exhibited the optical textures, devoid of PEO crystals (Figure 5b,c). The phase transition enthalpy (Δ*H*_LC-I_) of the homopolymer PM6BACP calculated for the sum of two peaks was 1.4 kJ/mol. On the other hand, the block copolymers PEO-*b*-PMA6BACP_46_ and PEO-*b*-PMA6BACP_106_ had Δ*H*_LC-I_ of 1.2 and 1.3 kJ/mol, respectively. The similar Δ*H*_LC-I_ values to the homopolymer indicate that the block copolymers possess LC properties derived from their LC blocks.

### 2.3. Microphase-Separated Structures and LC Layers

**Figure 6:**
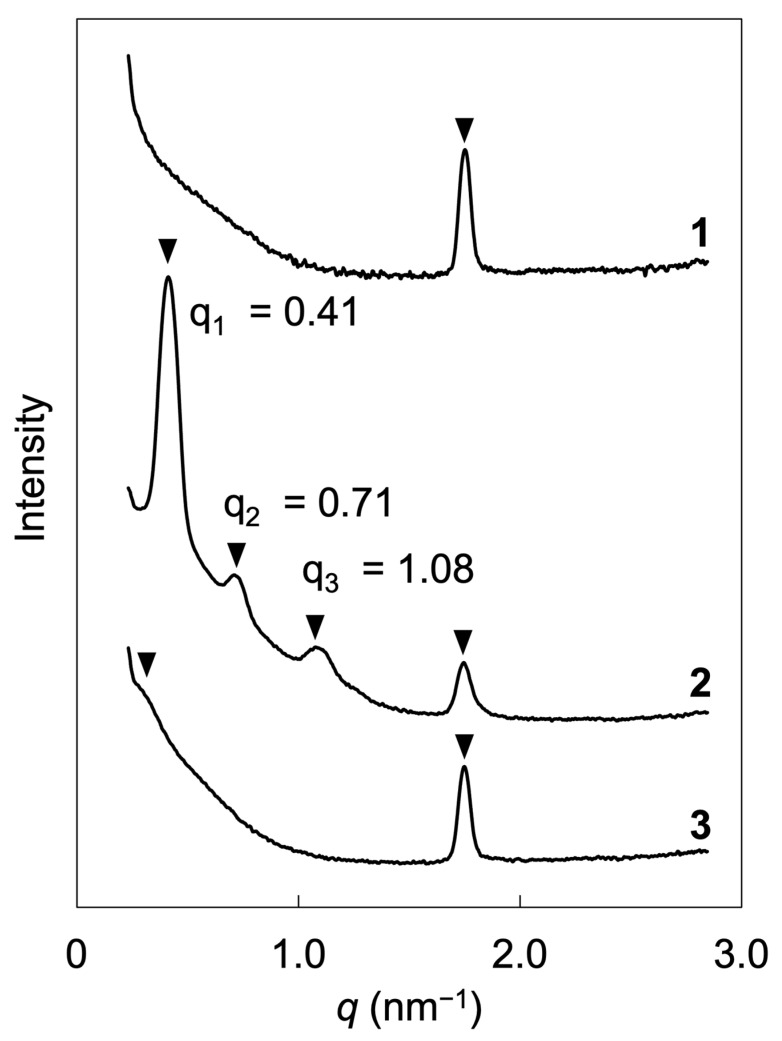
SAXS profiles of PM6BACP (**1**), PEO-*b*-PM6BACP_46_ (**2**), and PEO-*b*-PM6BACP_106_ (**3**).

Small-angle X-ray scattering (SAXS) measurements were undertaken to examine the microphase-separated structures of the block copolymers and LC layer structures within the homopolymer and block copolymers. Figure 6 presents the SAXS profiles obtained from bulk samples of the polymers, which were measured at room temperature subsequent to annealing under vacuum conditions at 130 °C for a duration of 1 h for PM6BACP and 24 h for PEO-*b*-PM6BACP_46_ and PEO-*b*-PM6BACP_106_. According to the DSC thermograms and POM observation, PEO blocks are supercooled, and the influence of crystallization on the SAXS measurements can be ignored. PM6BACP exhibited a sharp peak at 1.75 nm^−1^. Assuming a layered structure, the lattice period is estimated to be 3.6 nm, which is close to a *d* spacing of smectic A lamella structures of phenyl benzoate derivatives [44]. This result is consistent with the outcomes obtained from DSC and POM, affirming that PM6BACP displays a smectic phase and forms a layered structure. The peak was also observed for the block copolymers PEO-*b*-PM6BACP_46_ and PEO-*b*-PM6BACP_106_. As a result, LC molecules form smectic layers even within block copolymers. Furthermore, additional peaks were discernible in the case of the block copolymers. PEO-*b*-PM6BACP_46_ exhibited clear peaks at 0.41, 0.71, and 1.08 nm^−1^, where the ratio of the scattering vector of the primary peak to the second- and third-order peaks was 1:√3:√7. These findings imply the formation of microphase-separated structures featuring hexagonal cylinder arrangements [45]. From the *q* value of the primary peak, a lattice period of structures was calculated to be about 18 nm. Conversely, PEO-*b*-PM6BACP_106_ showed one broad peak at 0.39 nm^−1^. This broad peak indicates the lack of clear structure formation. The large molecular weight is generally advantageous for microphase separation. Therefore, PEO-*b*-PM6BACP_106_ is expected to induce more distinct microphase separation than PEO-*b*-PM6BACP_46_. The difference in results may be attributed to the factors to determine the morphology of the microphase-separated structures and the alignment of the LC molecules. Considering the fraction of PM6BACP in PEO-*b*-PM6BACP_106_, a microphase-separated structure with PEO sphere domains is expected to be stable. However, the PM6BACP block exhibits the smectic phase, which is more likely to stabilize PEO cylinders than spheres due to the higher-ordered alignment of the LC molecules. These two opposing effects may disrupt the organization of microphase-separated structures in PEO-*b*-PM6BACP_106_ with a large degree of polymerization of the LC polymer.

In a prior study, we explored the microphase separation of nematic LC block copolymers comprising PEO and polymethacrylate with a methoxy-terminated phenyl benzoate moiety as a side-chain mesogen. This study closely parallels the current one, primarily differing in the terminal group used [43]. The previous nematic LC block copolymer, having a similar degree of polymerization to PEO-*b*-PM6BACP_46_ with narrow molecular weight distribution, showed a primary peak with a second-order signal as a shoulder. Conversely, the block copolymer in the current study displayed clear evidence of microphase separation, as indicated by the presence of primary to third-order peaks in the SAXS profile. These findings strongly indicate the formation of a smectic phase, likely supported by the robust dipole moment of the cyano-terminated mesogens.

### 2.4. Alignment Behaviors of LC Polymers

**Figure 7:**
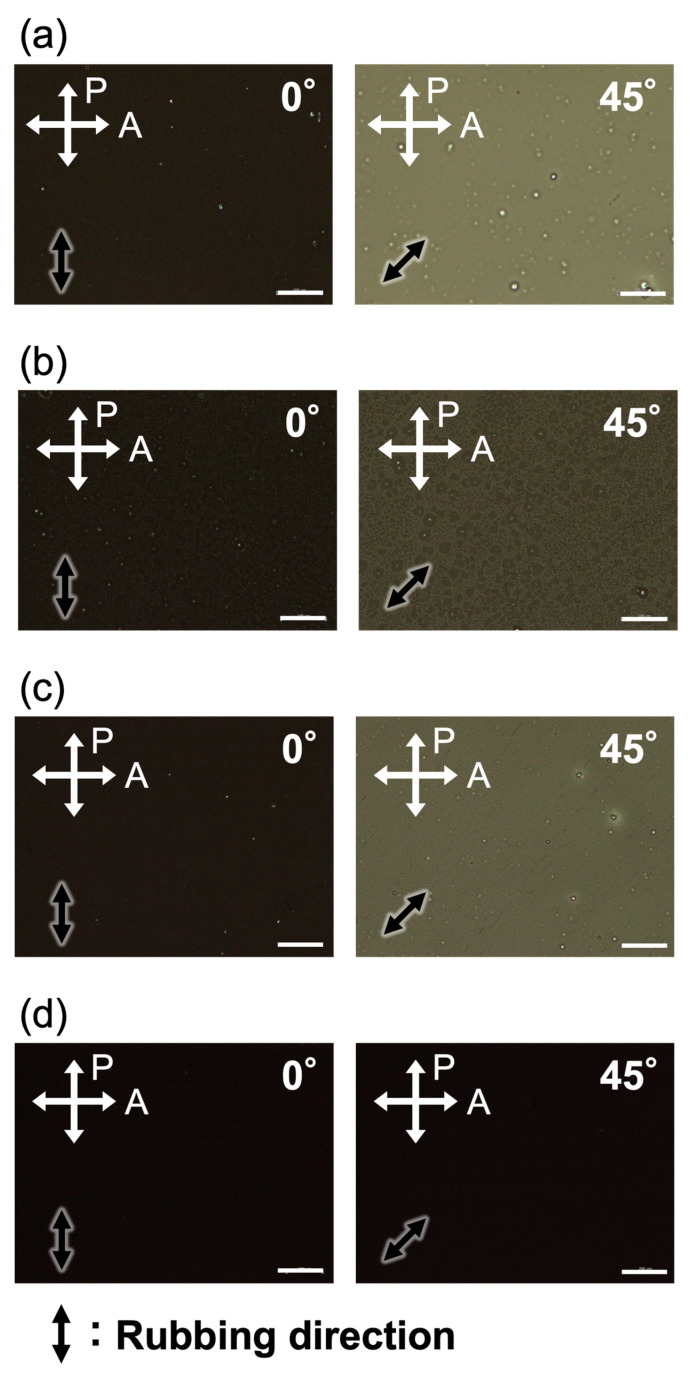
POM images of PM6BACP (**a**), PEO-*b*-PM6BACP_46_ (**b**), PEO-*b*-PM6BACP_106_ (**c**), and rubbed polyimide alignment layer (**d**). White crossed arrows show the direction of the polarizers. Black arrows show the rubbing direction. Scale bars, 200 µm.

The molecular alignment behavior of LC polymer films was evaluated. Thin films were prepared by spin-coating PM6BACP, PEO-*b*-PM6BACP_46_, and PEO-*b*-PM6BACP_106_ onto a glass substrate covered with a rubbed alignment layer. Following a 1 h annealing process at 130 °C, the optical characteristics of the polymer films were assessed through POM and polarized UV-visible absorption spectroscopy. The thicknesses of the films were 51, 31, and 47 nm, respectively (Table 2). POM observations of the thin films, conducted with crossed polarizers, revealed distinct contrast changes at every 45° rotation. The image darkened when the polarization direction aligned either parallel or perpendicular to the rubbing direction, a characteristic observed consistently in both the homopolymer and block copolymers (Figure 7a–c). On the other hand, the POM image of the rubbed polyimide alignment layer was completely dark regardless of the rotation angle (Figure 7d), confirming the optical anisotropy originates from the homopolymer and block copolymer thin films. The values of *R* were measured using a Berek compensator. Using the relation of *R* = *d*Δ*n*, where *d* is the film thickness and Δ*n* is birefringence, the values of Δ*n* were calculated to be 0.20, 0.19, and 0.19 for PM6BACP, PEO-*b*-PM6BACP_46_, and PEO-*b*-PM6BACP_106_, respectively.

**Figure 8:**
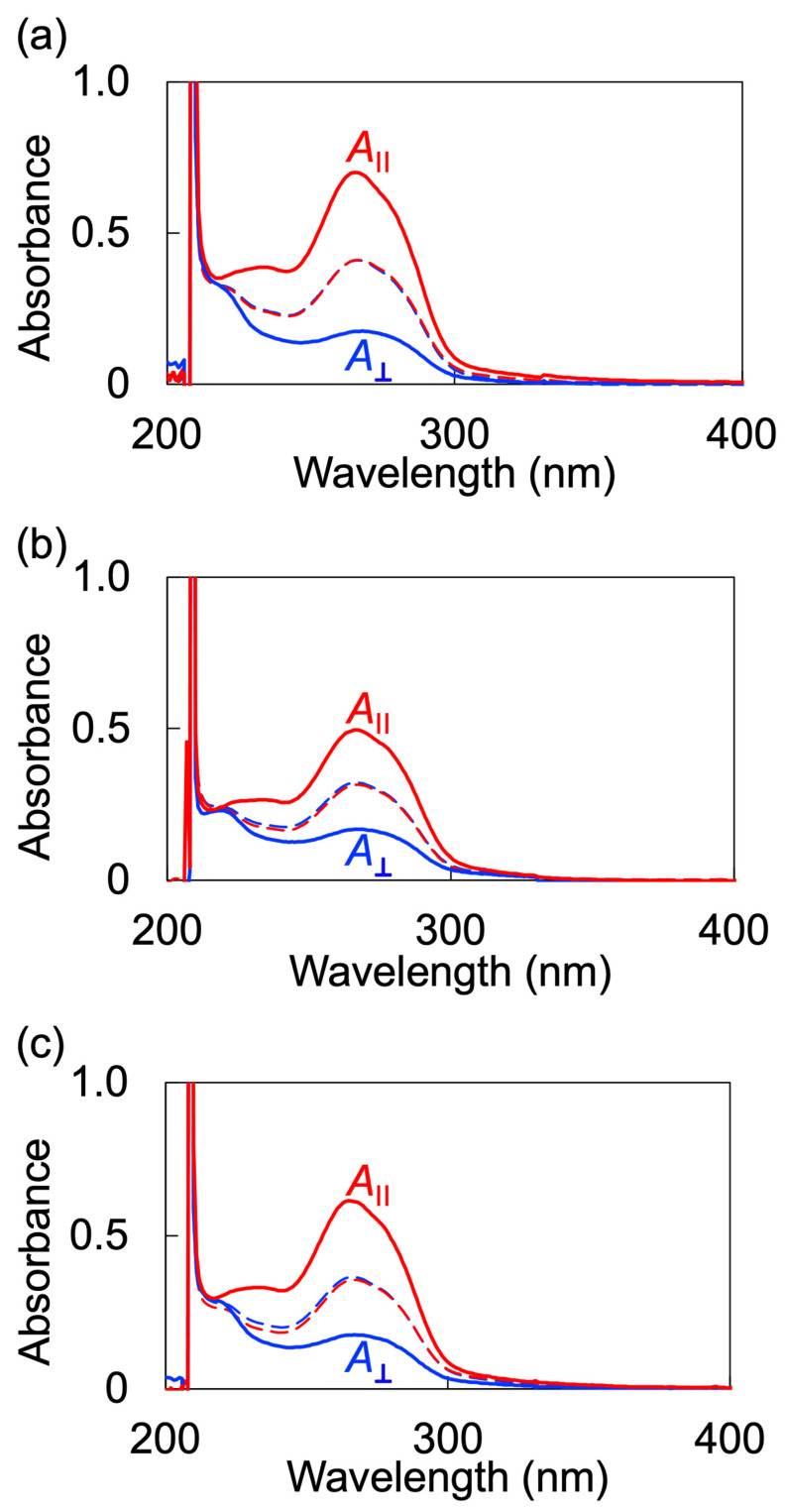
Polarized UV-vis absorption spectra of PM6BACP (**a**), PEO-*b*-PM6BACP_46_ (**b**), and PEO-*b*-PM6BACP_106_ (**c**). $A_{∥}$ and $A_{⊥}$ are absorbances parallel and perpendicular to the rubbing direction. Dashed and straight lines show the spectra before and after annealing, respectively.

For a more in-depth examination of the alignment behavior of LC polymers, we conducted measurements of polarized ultraviolet-visible (UV-vis) absorption spectra. We defined the absorbances parallel ($A_{∥}$) and perpendicular ($A_{⊥}$) to the rubbing direction, respectively. Polarized UV-vis absorption spectra of all samples exhibited no anisotropy before annealing (Figure 8a–c). Conversely, annealing led to increased absorbance parallel to the rubbing direction when compared to the perpendicular direction. The order parameter (*S*), serving as an indicator of the degree of in-plane alignment, was calculated using the following equation [46]:

(8) $$S=\frac{A_{∥}-A_{⊥}}{A_{∥}+2A_{⊥}}$$

The *S* value of the side-chain mesogen was calculated at 266 nm, where the wavelength of the maximum absorption of the phenyl benzoate exists. The *S* values for PM6BACP, PEO-*b*-PM6BACP_46_, and PEO-*b*-PM6BACP_106_ were 0.50, 0.40, and 0.45, respectively. Though the *S* values decreased as the proportion of non-LC PEO increased owing to the disruption of the alignment of the mesogen, the values are still close to the reported values for LC block copolymers with cyanobiphenyl moieties [41], which are well known as typical LC mesogens showing molecular alignments with high-order parameters. Therefore, it is suggested that the block copolymers consisting of cyano-terminated phenyl benzoate moieties have the potential to achieve long-range ordering with uniform alignment. Moreover, molecular alignment can facilitate the creation of highly oriented microphase-separated structures characterized by long-range ordering.

## 3. Materials and Methods

### 3.1. Materials

The monomer, 4-cyanophenyl 4-{[6-(methacryloyloxy)hexyl]oxy}benzoate (M6BACP), was provided by ENEOS Corp., Tokyo, Japan, and purified by silica gel column chromatography and recrystallization. The initiator, ethyl 2-bromoisobutyrate (E2BiB), was purchased from Tokyo Chemical Industry Co., Ltd., Tokyo, Japan, and used without further purification. The ligand, 1,1,4,7,10,10-hexamethyltriethylenetetetramine (HMTETA), was purchased from Sigma-Aldrich, Tokyo, Japan, and used without further purification. The catalyst, copper(I) chloride (Cu(I)Cl), was purchased from Sigma-Aldrich and used without further purification. The macroinitiator was synthesized from poly(ethylene glycol) methyl ether with *M*_n_ of 5000 (Sigma-Aldrich, Tokyo, Japan) and 2-bromoisobutyryl bromide (Tokyo Chemical Industry Co., Ltd., Tokyo, Japan) according to the previous report [25]. ATRP synthesized a homopolymer PM6BACP. M6BACP, E2BiB, HMTETA, and Cu(I)Cl were dissolved in anisole, degassed using five freeze–thaw cycles, and stirred at 80 °C for 20 h under a nitrogen atmosphere. The feed amount of the materials is summarized in Table 3. Following the reaction, the product was subjected to purification through an alumina column, with chloroform serving as the solvent. Subsequently, the product was reprecipitated initially with chloroform and hexane, and then once more with chloroform and diethyl ether. Block copolymers PEO-*b*-PM6BACP were synthesized using the identical procedure employed for the homopolymer PM6BACP with the use of M6BACP, macroinitiator, HMTETA, and Cu(I)Cl. The progress of polymerization and the rate of monomer conversion were confirmed by ^1^H NMR spectroscopy (Avance III, 400 MHz, Bruker Biospin, Bruker, Germany). The number-average molecular weight (*M*_n_) and polydispersity (*M*_w_/*M*_n_) were measured by size-exclusion chromatography (SEC) in a column (Shodex LF-804, Resonac Corp., Tokyo, Japan) equipped with a refractive index detector (Shodex RI101, Resonac Corp., Tokyo, Japan) and an ultraviolet light detector (UV-2075 plus, JASCO Corp., Hachioji, Japan). THF and polystyrene were used as the solvent and standards, respectively.

### 3.2. Film Preparation

**Figure 9:**
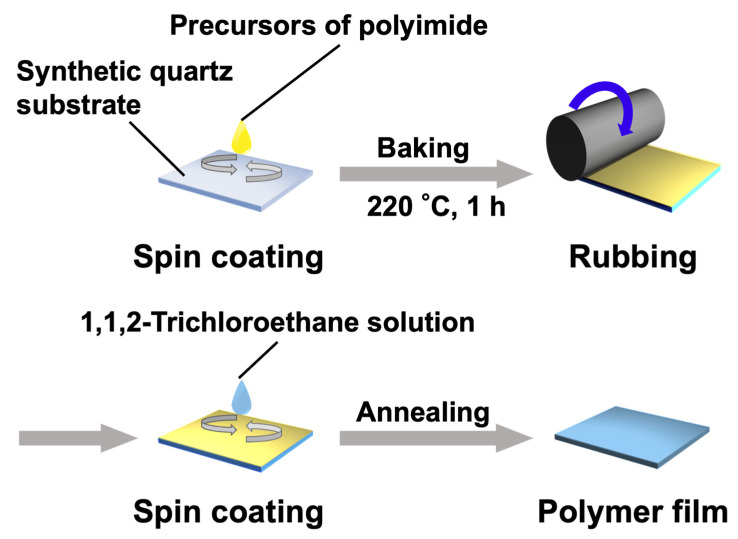
Schematic illustrations of the preparation of polymer films.

The synthetic quartz substrates underwent a cleaning process as outlined below. Initially, quartz glass substrates measuring 25 × 25 mm were ultrasonically cleaned with a neutral detergent for 15 min, twice with ultrapure water for 10 min, and with 2-propanol for 10 min. Following the cleaning process, the substrates were subjected to treatment using a UV-ozone cleaner (NL-UV42, Nippon Laser & Electronics Lab Co., Ltd., Nagoya, Japan) for 10 min. Next, the precursor solutions for the alignment layer (AL-1254, JSR Corp., Tokyo, Japan) were spin-coated on the cleaned glass substrates using a spin coater (MS-B100, Mikasa Co., Ltd., Tokyo, Japan) and heated at 60 °C for 1 min at a hot plate and 220 °C for 1 h in an oven (Figure 9). The resultant thin films were mechanically rubbed using a rubbing machine (MRG-100, EHC Co., Ltd., Tokyo, Japan). 1,1,2-trichloroethane solutions (1 wt%) containing PM6BACP and PEO-*b*-PM6BACP sufficient to cover the substrates were dropped, spin-coated, and annealed for 1 h at 130 °C at the isotropic phase temperatures. The rates of temperature increase and decrease were carefully controlled at 10 °C/min and 0.5 °C/min, respectively. Film thicknesses were measured with a stylus-type surface roughness meter (ET4000A, Kosaka Laboratory Ltd., Tokyo, Japan).

### 3.3. Characterization

The DSC measurement was performed using an Exstar DSC7000X differential scanning calorimeter (Hitachi High-Tech Corp., Tokyo, Japan). POM images were obtained by a BX 50 polarized optical microscope (Olympus Corp., Tokyo, Japan) equipped with a hot stage (HS82, Mettler-Tredo, Greifensee, Switzerland) and a Berek compensator (U-CBE, Olympus Corp., Tokyo, Japan). SAXS profiles were acquired using a Nano-Viewer (Rigaku Corp., Tokyo, Japan) equipped with a PILATUS 100k detector (Dectris, Baden, Switzerland). Polarized UV-vis absorption spectra were measured using a UV-vis absorption spectrophotometer (V-670, JASCO Corp., Hachioji, Japan).

## 4. Conclusions

In this study, we synthesized the monodisperse LC diblock copolymers with cyano-terminated phenyl benzoate moieties PEO-*b*-PM6BACP by ATRP. The evaluation of LC properties revealed that PEO-*b*-PM6BACP exhibits smectic LC phases. In PEO-*b*-PM6BACP with a narrow molecular weight distribution, hexagonal cylinder structures with a lattice period of 18 nm were formed by microphase separation. Thin films were fabricated and assessed for molecular alignment, revealing that the LC moieties exhibited unidirectional alignment along the rubbing direction in both PM6BACP and PEO-*b*-PM6BACP. The evident microphase separation, as revealed by SAXS analysis, and the confirmed molecular alignment behavior through POM and polarized UV-vis spectroscopy collectively point to the capability of LC block copolymers featuring cyano-terminated phenyl benzoate moieties to create well-ordered nanostructures.

## Figures and Tables

**Table 1:** Experimental conditions and characteristics of PM6BACP and PEO-*b*-PM6BACP synthesized by ATRP.

Sample	[M]_0_/[I]_0_	Conv (%) ^(a)^	DP		*M*_n_(calc) ^(d)^	*M*_n_(NMR) ^(e)^	*M*_n_(SEC) ^(f)^	*M*_w_*/M*_n_ ^(f)^	Weight Fraction of LC Block (%) ^(g)^
			PEO	PM6BACP					
PM6BACP	50	47	–	23 ^(b)^	9900	–	11,800	1.11	–
PEO-*b*-PM6BACP_46_	50	76	114	46 ^(c)^	20,700	24,000	19,200	1.09	78
PEO-*b*-PM6BACP_106_	100	84	114	106 ^(c)^	39,500	48,500	25,700	1.13	89

[Initiator]:[Cu(I)Cl]:[HMTETA] = 1:3:3. ^(a)^ Determined by ^1^H NMR according to Equation (1). ^(b)^ Calculated by ^1^H NMR according to Equation (2). ^(c)^ Determined by ^1^H NMR according to Equation (5). ^(d)^ Calculated by ^1^H NMR according to Equations (3) and (4). ^(e)^ Determined by ^1^H NMR according to Equation (6). ^(f)^ Determined by SEC. ^(g)^ Determined by ^1^H NMR according to Equation (7).

**Table 2:** Characterization of thin films of PM6BACP, PEO-*b*-PM6BACP_46_, and PEO-*b*-PM6BACP_106_.

Sample	*d* (nm)	Δ*n*	*S*
PM6BACP	51	0.20	0.50
PEO-*b*-PM6BACP_46_	31	0.19	0.40
PEO-*b*-PM6BACP_106_	47	0.19	0.45

**Table 3:** Feed amount of materials for the synthesis of PM6BACP, PEO-*b*-PM6BACP_46_, and PEO-*b*-PM6BACP_106_.

Sample	M6BACP (mg)	Initiator (mg)	Cu(I)Cl (mg)	HMTETA (mg)	Anisole (mL)
PM6BACP	310	3.0	1.5	3.5	2.5
PEO-*b*-PM6BACP_46_	310	75	1.5	3.5	2.5
PEO-*b*-PM6BACP_106_	610	75	1.5	3.5	2.5

## Data Availability

The authors confirm that the data supporting the findings of this study are available within the article.
